# From Behavior to the Built Environment: The Role of the Exposome on Cognitive Health

**DOI:** 10.1093/geroni/igaf122.1671

**Published:** 2025-12-31

**Authors:** Olivia Atherton

## Abstract

The exposome – the psychological, social, lifestyle, and physical environments where people live, connect, and work – plays an important role in supporting cognitive health across the lifespan. However, we know little about the mechanisms linking psychosocial, lifestyle, and built environment factors to cognitive health, as well as roles of the exposome across socioculturally-diverse populations. This symposium showcases innovative research that fills these gaps by leveraging longitudinal study designs (up to 14 years), multimethod data (e.g., self-report, geographical linkages, cognitive batteries), lifespan perspectives (from adolescence through older adulthood), and socioculturally-diverse populations (including samples of Mexican-origin and Latino individuals, and an adoption/twin study). First, Dr. Emily Willroth uses data from a 14-year longitudinal study of Mexican-origin adults to examine how well-being (i.e., life satisfaction, optimism) is related to modifiable dementia risk factors and cognitive health. Second, Dr. Graciela Muniz Terrera uses longitudinal data from a Latino sample to demonstrate how social cohesion and loneliness are associated with cognitive function and trajectories. Third, Dr. Chandra Reynolds uses longitudinal data from an adoption-twin study to demonstrate the role of neighborhood Cognability on cognitive performance in midlife, while also considering demographic, developmental, and neighborhood covariates. Finally, Dr. Olivia Atherton uses geographical linkages to the Health and Retirement Study to characterize discrepancies between health behavior engagement and built environment opportunities, as well as the impact of behavior-opportunity gaps on cognitive health. We conclude with a general discussion of future directions in understanding the role of the exposome on cognitive health and healthy aging in place.

